# Evaluation of Accelerometer-Based Fall Detection Algorithms on Real-World Falls

**DOI:** 10.1371/journal.pone.0037062

**Published:** 2012-05-16

**Authors:** Fabio Bagalà, Clemens Becker, Angelo Cappello, Lorenzo Chiari, Kamiar Aminian, Jeffrey M. Hausdorff, Wiebren Zijlstra, Jochen Klenk

**Affiliations:** 1 Department of Electronics, Computer Science and Systems, University of Bologna, Bologna, Italy; 2 Department of Clinical Gerontology, Robert-Bosch-Hospital, Stuttgart, Germany; 3 Laboratory of Movement Analysis and Measurement, Ecole Polytechnique Fédérale de Lausanne (EPFL), Lausanne, Switzerland; 4 Tel Aviv Sourasky Medical Center, Laboratory for Gait and Neurodynamics, Movement Disorders Unit and Department of Physical Therapy, Sackler Faculty of Medicine, Tel-Aviv University, Tel-Aviv, Israel; 5 Center for Human Movement Sciences, University Medical Center Groningen, Groningen, The Netherlands; Cardiff University, United Kingdom

## Abstract

Despite extensive preventive efforts, falls continue to be a major source of morbidity and mortality among elderly. Real-time detection of falls and their urgent communication to a telecare center may enable rapid medical assistance, thus increasing the sense of security of the elderly and reducing some of the negative consequences of falls. Many different approaches have been explored to automatically detect a fall using inertial sensors. Although previously published algorithms report high sensitivity (SE) and high specificity (SP), they have usually been tested on simulated falls performed by healthy volunteers. We recently collected acceleration data during a number of real-world falls among a patient population with a high-fall-risk as part of the SensAction-AAL European project. The aim of the present study is to benchmark the performance of thirteen published fall-detection algorithms when they are applied to the database of 29 real-world falls. To the best of our knowledge, this is the first systematic comparison of fall detection algorithms tested on real-world falls. We found that the SP average of the thirteen algorithms, was (mean±std) 83.0%±30.3% (maximum value = 98%). The SE was considerably lower (SE = 57.0%±27.3%, maximum value = 82.8%), much lower than the values obtained on simulated falls. The number of false alarms generated by the algorithms during 1-day monitoring of three representative fallers ranged from 3 to 85. The factors that affect the performance of the published algorithms, when they are applied to the real-world falls, are also discussed. These findings indicate the importance of testing fall-detection algorithms in real-life conditions in order to produce more effective automated alarm systems with higher acceptance. Further, the present results support the idea that a large, shared real-world fall database could, potentially, provide an enhanced understanding of the fall process and the information needed to design and evaluate a high-performance fall detector.

## Introduction

Despite extensive preventive efforts, falls continue to be a major source of morbidity and mortality among older adults. Falls often lead to serious injuries such as hip fractures, hospitalization and death. Even when no serious injury occurs, the resultant fear of falling and self-imposed restrictions in mobility and function may contribute to nursing home admission [1] and lead to a loss of personal autonomy that directly affects the quality of life of subjects.

Falls among older people remain a very important public healthcare issue.

Real-time detection of falls allows for the immediate communication of these adverse events to a telecare center so that medical assistance can be supplied quickly. Such assistance is needed to promote the sense of security of older adults, especially among those who are living alone, and to reduce fear of falling and the subsequent negative impact of falls. Indeed, one of the serious consequences of falling is the “long-lie” condition, where a faller is unable to get up and remains on the ground for several hours. “Long-lies”, and falls, in general, are associated with social isolation, fear of falling, muscle damage, pneumonia, pressure sores, dehydration and hypothermia [2]–[5]. Half of the elderly people who experience a ‘long-lie’ die within 6 months [6], even if no direct injury from the fall has occurred. The ‘long-lie’ occurs in more than 20% of elderly people admitted to hospital as a result of a fall [7] and up to 47% of non-injured fallers are unable to get up off the floor without assistance [8]. Detection of a fall, either through automatic fall detection or through a personal emergency response system, might reduce the consequences of the ‘long-lie’ by reducing the time between the fall and the arrival of medical attention [9]. If an older person living alone experiences a fall at home, he or she may not be able to get to the phone or press an alarm button due to sustained injuries or loss of consciousness [10]. Moreover, some elderly people do not activate their personal emergency response systems, even when they have the ability to do so [11].

For these reasons, a variety of different methods were developed over the last decade to automatically detect falls. These have been based on video-cameras [12]–[17], acoustic [18]–[21] or inertial sensors [22]–[44], and mobile phone technology [45]–[47].

Several of these studies focused on the monitoring of activities of daily living (ADL) and fall detection using wearable sensors. Compared to traditional movement analysis systems, wearable sensors offer advantages in terms of cost, size, weight, power consumption, ease of use and, most importantly, portability. With wearable sensors, data collection is no longer confined to a laboratory environment, thus leading to ubiquitous health monitoring.

Many different approaches have been explored to solve the fall detection problem using only accelerometers or an inertial measurement unit (both gyroscopes and accelerometers) [28]–[35], [38]–[40]. The analysis of accelerometer and/or gyroscope outputs allows for detecting specific events, such as voluntary (e.g., walking, sitting, lying) or involuntary (e.g., fall) activities of daily living, based on statistical or threshold-based algorithms.

The inertial sensor-based fall detection algorithms usually provide: i) a definition of a set of parameters related to the accelerometer and gyroscopes outputs, used for the characterization of the movement, ii) impact detection, using a threshold-based method, iii) orientation detection, e.g., using the vertical accelerometer output or angular rate measurements, and iv) fall alarm, which occurs when all the test conditions are true.

Published algorithms have generally been tested only on simulated falls. Most authors have used simulations with healthy volunteers [29], [30], [32]–[34], [38], [41], [42], [44], [45] or martial arts students [28] as a surrogate for real-world falls [48]. To the best of our knowledge, there is a lack of published inertial measurement-based real-world fall data of older people measured in a real-world environment.

Although the rate of falls is quite high (approximately 30% of persons over 65 years fall at least once per year), it is very difficult to capture real-world fall data. This largely is a result of the relatively short measurement intervals allowed by commercially available sensors. As an example, to capture 100 real-world falls, it would be necessary to record approximately 100,000 days of physical activity (300 person years). If the battery lifetime is limited to 10 days, 10,000 measurement cycles would be needed. Additionally, compliance problems may arise with long measurement periods. As far as we know, most international studies have failed to gather sufficient numbers of fall events. Recently, Kangas *et al.* [49] collected acceleration data of 5 real-world falls during a six-month test period in older people.

To address the challenges of capturing real-world falls, we began to collect acceleration data during a number of real-world falls as part of a European project (SensAction-AAL) that studied a population with a high-risk of falling. Based on these data, a recent study [50] compared acceleration signals, measured using a tri-axial accelerometer placed on the waist of the subjects, from simulated falls and these real-world falls and found large differences between them, even though a relatively simple example of falling backward to the ground was selected.

Several problems are associated with the simulation approach including the anticipation of the volunteer that a fall will occur and the choice of the floor material to reduce the impact of the falls for safety reasons. These findings underline the importance of gathering real-world fall data for designing accurate algorithms.

With the limitations of simulated falls in mind, the aim of the present study is to benchmark, for the first time, the performance of 13 different published algorithms as applied to the database of 29 real-world falls collected during the SensAction-AAL project. In order to compare the performance in the same test conditions as our real-world fall data, only algorithms based on waist or trunk accelerometer measurements were investigated. Algorithms based on gyroscopes measurements or on more than one sensor are not considered in this paper.

## Materials and Methods

### 1. The real-world fall database

Acceleration signals of 32 falls from 15 subjects were collected during the SensAction-AAL project and clinical routine assessments. 30 falls from 9 subjects (7 women, 2 men, age: 66.4±6.2 years, height: 1.63±8.68 *m*, weight: 77.2±11.5 *kg*) were recorded within a cross-sectional study of patients suffering from progressive supra-nuclear palsy (PSP) [51] and from an intervention study to investigate the feasibility of audio-biofeedback to improve balance [52]. PSP is an atypical Parkinson's syndrome with a prevalence of 5 per 100,000 [53]. Postural instability and falls are common and are the most disabling features of the disease [54], [55]. A 48-h activity measurement was conducted on 29 subjects as part of the assessment in the cross-sectional study and during days without intervention. A fall was defined as “*an unexpected event in which the participant comes to rest on the ground, floor, or lower level*” [56]. Patients or their proxies reported the time, the place and the circumstances of the falls.

Two additional falls were recorded from one subject within a cross-sectional study in community-dwelling older people. All of these falls were recorded during daily physical activity measurement using an ambulatory device based on accelerometers (Dynaport® MiniMod, McRoberts, The Hague, NL). For the sake of the present study, for each fall, we extracted, from the 24 hour recording, a 60 second time-window centered around the fall event. The falls were characterized with respect to location, pre-fall phase, fall direction, and impact spot (Table 1). The MiniMod®, composed of a tri-axial seismic acceleration sensor (LIS3LV02DQ STMicroelectronics, Agrate Brianza, Italy), was fixed by a belt at the lower back. The orientation of the axes are x = vertical, y = medio-lateral (left/right), and z = anterior-posterior (forward/backward). The sensor has a resolution of 12 bit and a sampling frequency of 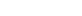. The published fall detection algorithms were usually based on measurements carried out by accelerometers with a sampling frequency varying from 50 *Hz* to 250 *Hz* and a range of ±10 *g* or ±12 *g*. We recorded 14 falls with a sensor's range of ±6 *g*, the remaining 18 falls with a sensor's range of ±2 *g*. When the acceleration exceeds the threshold ±2 *g*, the so-called “clipping effect” (or saturation) produces a cut-off of the signal. Since this could affect the results of the analysis, three falls that show saturation effects are not included in the analysis. Therefore, the total number of falls considered in this study was 29. Raw data were stored for off-line analysis on a SD card.

**Table 1:** Description of real-world falls (*n* = 32).

	Number of falls per condition
Location	Indoor (*n* = 30), outdoor (*n* = 2)
Activity before the fall	Standing (*n* = 16), walking forward (*n* = 8), walking backward (*n* = 1), sit-to-stand (*n* = 5), stand-to-sit (*n* = 2)
Reported direction of fall	Forward (*n* = 8), backward (*n* = 18), sideward (*n* = 6)
Impact spot	Floor (*n* = 23), against wall/locker before hitting the floor (*n* = 4), bed/sofa (*n* = 4), desk (*n* = 1)

### 2. The algorithms

The algorithms used are summarized here; additional details can be found in the literature [28]–[32]. Table S1 summarizes the parameters, thresholds and the phases of a fall event that are considered: beginning of the fall, falling velocity, fall impact and orientation after the fall. The outputs of the tri-axial accelerometer are 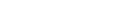, with *k = 1,…,n* where *n* is the number of samples.

Chen *et al.* [28] used a tri-axial accelerometer worn on the waist of two martial arts students, who performed some common fall motions over 10 trials. If the root sum vector (*SV*) of the three squared accelerometer outputs exceeds a threshold, it is possible that a fall has occurred (IMPACT DETECTION). Additionally, the orientation is calculated over 1 second before the first impact and 2 second after the last impact using the dot product of the acceleration vectors (CHANGE IN ORIENTATION). The angle change that constitutes a change in orientation can be set arbitrarily based on empirical data, as suggested by the authors. We set this threshold to 20° in order to have the best sensitivity and specificity. No results are reported in the paper, but the authors point out the benefits due to the evaluation of change in orientation.

Kangas *et al.* [29] attached a tri-axial accelerometer to the waist, wrist and head of three healthy middle-aged volunteers, who performed three standardized types of falls (forward, backward, and lateral) towards a mattress. Examples of activities of daily living (ADL) were collected from two healthy subjects, representing dynamic activities (e.g., walking, walking on the stairs, picking up objects from the floor). Four different detection algorithms, Kangas1a to Kangas1d, with increasing complexity were investigated. The thresholds are related to the waist measurement. These four algorithms had in common IMPACT DETECTION + POSTURE MONITORING. They were based on the detection of the impact by threshold on the sum vector (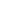), the dynamic sum vector (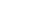) related to the high-pass filtered (HPF) accelerometer outputs, the sliding sum vector (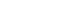) and the vertical acceleration (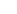), respectively, followed by monitoring of the subject's posture. The posture was detected 2 seconds after the impact from the low-pass filtered (LPF) vertical signal, based on the average acceleration in a 0.4 second time interval, with a signal value of 0.5 *g* or lower considered to be a lying posture.

Two further algorithms, Kangas2a and Kangas2b, were considered from Kangas et al. [29] based on START OF FALL + IMPACT DETECTION + POSTURE MONITORING. These algorithms detected the start of the fall by monitoring 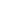 lower than a threshold of 0.6 *g*, followed by the detection of the impact within a time frame of 1 *s* by a threshold value of 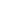 or 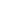, followed by posture monitoring.

**Figure 1:**
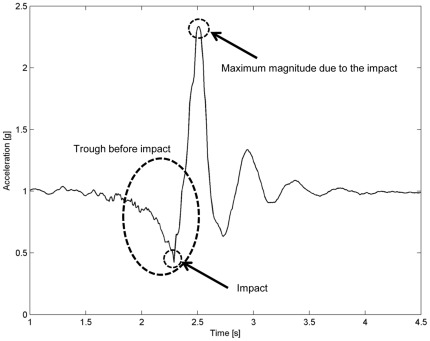
Prototypical acceleration sum vector of a fall. This real-world example illustrates components that are common to many falls.

Three further algorithms, Kangas3a to Kangas3c, based on START OF FALL + VELOCITY + IMPACT DETECTION + POSTURE MONITORING were considered from [29]. These algorithms detected the start of the fall, followed by detection of the velocity 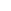 (calculated by integrating the area of 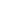 from the trough (see Fig. 1), at the beginning of the fall, until the impact, where the signal value is lower than 1 *g*) exceeding the threshold, followed by the detection of the impact within a time frame of 1 *s* by a threshold value of 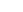 or 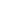, followed by posture monitoring. The fall detection sensitivity, declared by the authors [29], of the different eight algorithms at the waist varied from 76% to 97% and the specificity was 100%.

Bourke *et al.* [30] fixed two tri-axial accelerometers to the trunk (at the sternum) and the thigh. Ten young subjects were involved in simulated falls onto large crash mats. Ten community-dwelling elderly subjects performed ADL in their own homes (e.g., sit to stand, lying, walking). In these algorithms (Bourke1a and Bourke1b), the *SV* of the three signals was evaluated from the sternum and thigh accelerometer outputs and a fall was detected when the *SV* is over the upper (UFT) threshold (3.52 *g*) or under than the lower (LFT) threshold (0.41 *g*). Declared specificity is 100% for the upper threshold and 91.25% for the lower threshold, related to the trunk sensor. In this paper, as suggested by the authors [30], the thresholds were set according with the falls database. The UFT and LFT were set at the level of the smallest magnitude upper fall peak (Bourke1a) and at the level of the biggest magnitude lower fall peak (Bourke1b), respectively. Based on the accelerometer data of the 29 falls, we set the two thresholds to 1.79 g (UFT) and 0.73 g (LFT). Exceeding any individual limit would indicate a fall.

Bourke *et al.* [31] developed a second fall detection system using a tri-axial accelerometer to detect impacts. The algorithm (Bourke 2), considered the *SV* of the accelerometer outputs, and monitor posture, assuming a lying posture if the vertical accelerometer signal value is between −0.5 *g* and 0.5 *g*. The sensor was attached to a custom designed vest. Two teams of 5 elderly subjects tested the algorithm. Over 833 hours of monitoring, no actual falls were recorded, although the system registered a total of 42 false alarms (i.e., false positives).

Recently, Bourke *et al.* [32] evaluated 21 fall-detection algorithms of varying degrees of complexity for a waist-mounted accelerometer based system. The algorithms were tested against a comprehensive data-set recorded from 10 young healthy volunteers performing 240 simulated falls and 120 ADL and 10 elderly healthy volunteers performing 240 scripted ADL and 52.4 waking hours of continuous unscripted normal ADL. Here, we evaluated the algorithm (Bourke3) VELOCITY+IMPACT+POSTURE that achieved 100% sensitivity and specificity and with the lowest false-positive rate (0.6 false positive per day) when applied to simulated falls and tested it on the real-world falls database. The algorithm is based on the detection of the four distinct phases of a fall [48] (pre-fall, critical phase, post-fall phase and recovery) when the SV exceeds the LFT (0.65 *g*) and the UFT (2.8 *g*) thresholds. Two temporal features and their related thresholds are considered: the falling-edge time, 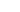, is from the SV signal last going below the LFT until it exceeds the UFT (threshold set to 600 *ms*), and the rising-edge time, 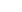, is the last time when the LFT is exceeded until the UFT is exceeded (threshold set to 350 *ms*). The vertical velocity is further considered as an indicator of a fall when it overcomes the threshold 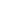 (−0.7 *m/s*). It is evaluated through the numerical integration of the SV signal with the gravity component subtracted. The post-fall posture is determined taking the dot product of the gravity vector 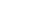 and the current gravity vector estimated relative to the body segment 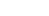. Lying is detected if the waist posture, 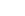, from t+1 s to t+3 s exceeds 60° for more than 75% of the duration.

As summarized in Table S1, the *SV* is a common feature among all the algorithms. An example of prototypical signal of the *SV* is shown in Fig. 1. The signal reflects a forward real-world fall in which the subject fell directly on the floor while bending to pick up an object. The typical trough before the impact, the impact and the maximum magnitude due to the impact are also indicated.

The 29 accelerometer fall recordings were used to test the performance of the algorithms in terms of sensitivity (SE, percentage of falls correctly detected as such). Further signal analysis was performed in order to evaluate the specificity (SP, percentage of ADL correctly identified as non-falls).

Previous studies tested the specificity of ADL performed in the laboratory environment by the same subjects who simulated falls (generally healthy young subjects) or community-dwelling elderly subjects. These data could be biased, since subjects are forced to perform activities, which are typically spontaneous. To avoid biased results for specificity, we extracted ADL based on the individual physical activity recordings from each subject excluding the 60 second fall-time-windows. The remaining observation time was also separated into 60 second time-windows.

The recordings of 8 of the 15 fallers were carried out using the sensor with range ±2 *g* and therefore were excluded from the specificity evaluation. We collected, for the remaining 7 subjects, 168 *h* of accelerometer recordings, i.e., 10,050 time-windows of 60 seconds (the 29 time-windows related to falls were excluded). These time windows could be related to resting periods. In all these cases, the fall detection algorithms correctly identify 100% of ADL as not-falls. Thus, the SP will show high values because of the high number of time windows with inactivity included in the analysis. According to these considerations, the time windows related to resting periods were excluded and those related to activity periods were considered in the study according with a simple procedure. We assumed that an activity is performed if the dynamics of the signal (the difference between the maximum and the minimum value) in a 60 second time window overcomes a fixed threshold TH. This was selected from the following steps:

the difference 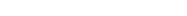 (i = 1,…, 10,050) was evaluated from the accelerometer outputs for each of the 10,050 time-windows;
the 10,050 time-windows were tested by the 13 algorithms;
if the *k*-th time window was wrongly identified as fall, the value 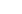 was allocated in a vector **M**;
after testing the 13 algorithms, the minimum element of **M** was considered as the threshold TH for discriminating resting from activity periods.

All the time-windows with 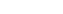 were considered as ADL and thus selected for the analysis. The threshold evaluated by the procedure was TH = 1.01 *g*. The total number of time-windows considered was 1,170.

The accuracy (ACC, the ratio between the number of correct assessments, falls and ADL, and the number of all assessments), the positive predictive value (PPV, the probability that a time window with a positive test result, fall detected, really does have the condition for which the test was conducted) and the negative predictive value (NPV, the probability that a time window with a negative result, fall undetected, really does have the condition for which the test was conducted) were evaluated for each algorithm.

Moreover, the performance of the tested algorithms were evaluated on 24 hour accelerometer recordings for three of the PSP fallers, in order to evaluate the number of false alarms (ADL detected as falls) generated by the different algorithms.

Data analysis was performed using MATLAB 7.9.0 (R2009B).

## Results

**Figure 2:**
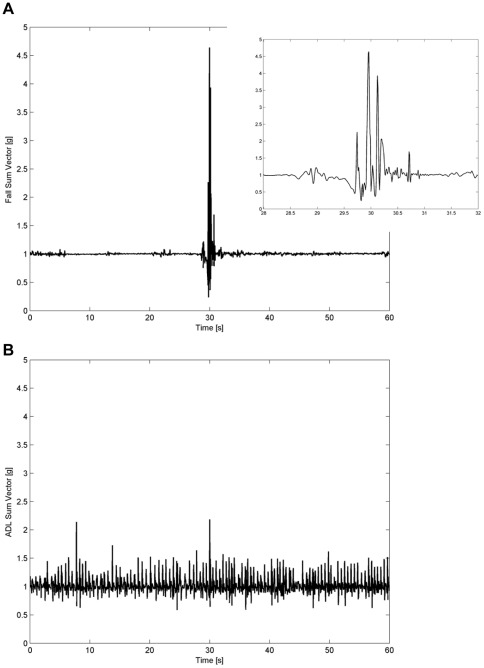
Sum vector of (a) backward fall and detail and (b) example of selected ADL (walking).

In order to show an example of real-world fall signals, the sum vector of a backward fall and its detail is reported in Fig. 2(a). The sum vector related to one of the randomly extracted ADL is shown in Fig. 2(b).

**Figure 3:**
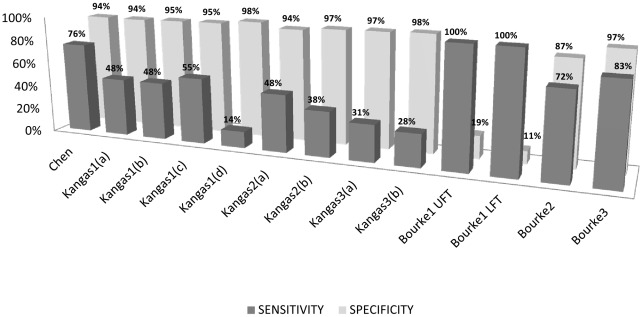
Sensitivity and Specificity for the tested algorithms.

The SE and SP of the tested algorithms for fallers are shown in Fig. 3. The SP is over 94% for all the algorithms, except for Bourke2 and Bourke1(a, b), which have the best performance in terms of SE (the thresholds are set to hit this mark) and the worst in terms of SP, as one would expect. The SE is low (SE = 57.0%±27.3%, maximum value = 82.8%). Although a trade-off is achieved with the Chen and Bourke3 algorithms (SE = 75.9%–82.8%, SP = 94.2%–96.7%, respectively), the results are considerably worse than those previously reported in laboratory environments.

The ACC, the PPV and the NPV are reported in Table 2 for each algorithm.

**Table 2:** Accuracy (ACC), positive (PPV) and negative (NPV) predictive values of the tested algorithms.

Algorithm	ACC [%]	PPV [%]	NPV [%]
*Chen*	93,7	24,4	99,4
*Kangas1a*	92,9	16,7	98,7
*Kangas1b*	93,7	18,9	98,7
*Kangas1c*	94,5	23,2	98,8
*Kangas1d*	96,4	18,2	97,9
*Kangas2a*	93,3	17,7	98,7
*Kangas2b*	95,3	22,4	98,4
*Kangas3a*	95,8	23,1	98,3
*Kangas3b*	96,7	29,6	98,2
*Bourke1a*	21,3	3,0	100,0
*Bourke1b*	13,0	2,7	100,0
*Bourke2*	86,8	12,3	99,2
*Bourke3*	96,3	38,1	99,6

**Figure 4:**
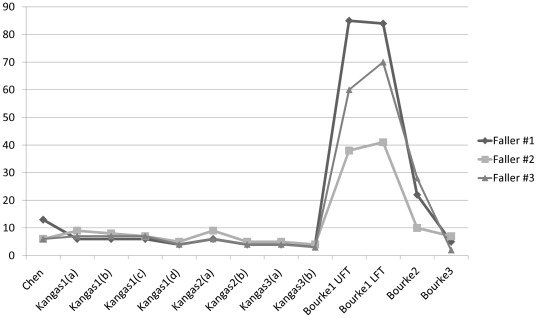
False alarms generated in 24 *h* recordings for three fallers.

The number of false alarms generated in 24 hours is shown in Fig. 4 for three fallers. Bourke1(a, b) shows the highest number of false alarms, although it has the maximum value of sensitivity. Kangas' algorithms generated less than 9 false alarms, but sensitivity was lower than 55% (Fig. 3).

## Discussion

The aim of this study was to compare different accelerometer-based fall detection algorithms on a database of real-world falls. Consistent with our previous work which demonstrated marked differences between real-world and simulated falls [50], we find that algorithms that were successful at detecting simulated falls did not perform well when attempting to detect real-world falls.

To our knowledge, no other studies in the literature have evaluated fall detection algorithms based on a relatively large dataset of real-world falls. Fifteen older patients (age 67±18 years) assessed as having a high risk of falling were involved in an 18-day study [31]. Unfortunately only four falls were recorded and the data were not analyzed. Recently, Kangas *et al.* [49] collected accelerometer data for 5 real-world falls during a 6 month test period in older adults and compared some features (SV, pre-fall velocity) of real-world falls with simulated falls. They suggested that there are important differences between real and simulated falls.

Based on a data-set with recorded real-world falls, our study evaluated thirteen accelerometer-based algorithms for fall detection which have been previously evaluated on simulated falls only. SE and SP of these algorithms (Fig. 3) show how the sensitivity and specificity obtained by the authors (often declared to be 100%) are different when the algorithms are tested on real-world falls.

By analyzing the main drawbacks of the presented algorithms, we noted that several factors affect the difference between simulated and real-world falls. Thresholds are usually calibrated on simulated fall signals and are not suitable for real-world fall signals. For instance, the SV is considered to be a feature for impact detection in all the presented algorithms, but each author used a different threshold to detect the impact. Our results are consistent with the considerations of Kangas [49] who found that some fall phases detected in experimentally simulated falls were not detectable in acceleration signals from heterogeneous real-world falls.

Nevertheless, all the algorithms have low computational cost and low-complexity, allowing them to be easily implemented in a microcontroller for real-time applications. This approach is commendable because it helps to increase the security of the subject, hopefully reducing the number and severity of falls. The use of the sum vector of the three accelerometer outputs as the main parameter provides robustness against the incorrect position of the sensor.

Chen's algorithm [28] provides a good trade-off in terms of SE (76%) and SP (94%). Since the high threshold for impact detection allows for reduction of false positives, some “low-magnitude” falls are not detected due to their maximum peak values. Despite the efforts of the authors to pay attention to orientation change, this parameter does not provide an optimal discrimination between real-world falls and ADL. Since we set a low angle orientation threshold, in many conditions the subject's orientation does not show a significant change before and after the fall (e.g., falling on the knees).

Kangas *et al.* [29] investigated three different algorithms with increasing complexity. Since the threshold values allow detection of most impacts, the posture monitoring test fails against several types of falls. The LPF vertical signal rarely reaches values under 0.5 *g*, which is considered to be a lying posture [35], [36]. In our falls database, subjects who fell on their buttocks, knees, or against a table or the walker, did not lie on the floor. However, the lying posture detection is very important to detect falls in which the subject lies on the floor for a long time. Moreover, according to the SE values shown in Fig. 3, the more complex the algorithm, the more assumptions about thresholds have to be satisfied and so the less likely it is to detect a fall. As discussed previously, these thresholds are calibrated on simulated fall signals and their values should be reconsidered on real-world fall signals. The algorithms related to the vertical acceleration 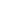 (Kangas1d, Kangas2b, Kangas3b) provide the lowest sensitivity values due to the high threshold set for this parameter. Kangas3(a, b) show the worst results: the velocity before the impact is often lower than the predetermined thresholds.

Results of Bourke's algorithm [30] require additional discussion. The authors suggest setting the two thresholds according to the falls database in order to have 100% of SE. Nevertheless, the Bourke1(a, b) algorithms provide the worst results in terms of SP (19.3% and 11.9%, respectively). This offline method is not recommended for several reasons. First, the two thresholds, UFT (1.79 *g*) and LFT (0.73 *g*), are set according to the real-world fall database and therefore have to be tuned every time a new fall occurs. Consequently, the predictive ability of the fall detection algorithm is impaired: if the fall detector is used in real-life conditions, falls with a maximum peak lower than UFT or the minimum peak greater than LFT are not detected. The Bourke1b algorithm, based on the LFT algorithm, provides the lowest SP. The majority of real-world falls we collected provide a trough before the impact related to the free-fall phase. However some ADL (e.g., sitting on a chair or on a bed) show values of the sum vector lower than the LFT = 0.73 *g*, due to the phenomenon of weightlessness. This explains why the lowest specificity was found for Bourke's algorithm. Moreover, as shown in Fig. 4, the high number of false alarms during 24 *h* recordings, from 22 to 85 for the three fallers for Bourke1a, and from 27 to 84 for Bourke1b, is unacceptable (more than 2 false alarms per hour). The major reason for failure is rejection by monitoring services due to a high number of false alarms [57], [58]. This weak point is more evident in the Bourke LFT algorithm as compared to the other algorithms, since these have fewer than ten false alarms.

The second algorithm suggested by Bourke *et al.* [31] provides results similar to Chen's algorithm. Since the threshold, which is the same as Bourke1b, ensures detection of several impacts, the SP is higher than Bourke1(a,b) because the algorithm provides posture monitoring after fall. The subject's “long-lie” condition (vertical accelerometer signal value between −0.5 *g* and 0.5 *g*) allows increasing the SP but this did not occur in all falls. The Bourke2 algorithm emphasizes the drawbacks of the Bourke1 algorithms, which are based on thresholds lacking considerations of posture monitoring after a fall. Adding information about posture after the impact can improve the results in terms of SP. As shown in Fig. 4, the number of false alarms is reduced threefold from Bourke1 (mean of 61–65 false alarms) to Bourke2 (20 false alarms).

Bourke's recently proposed algorithm (Bourke3) [32] provides the best trade-off in terms of SE (83%) and SP (97%) but the results are still different from those obtained by the authors on their simulated-falls database (100% sensitivity and specificity). The algorithm fails to detect falls with low impact magnitude (mainly forward falls, falling on bed/sofa, against the wall) which are common, since more than half of all in-patient falls in elderly people in acute care settings occurred at bedside, during transfer or while getting up [59], [60]. Moreover, Kangas *et al.* [49] also found differences between simulated and real-world falls on beds in terms of low impact magnitude. Despite of this, the number of false alarms is considerably lower than with other Bourke's algorithms (about 5 false alarms per day).

Table 2 provides results related to PPV and NPV, which are usually stable characteristics of diagnostic tests when the prevalence of disease is high among the population of interest (in this study prevalence of disease is equivalent to fall risk). The same diagnostic test will have varying predictive values in different populations. As mentioned in the Methods section, the recorded ADL, used for testing the algorithms, are related to the PSP and geriatric rehabilitation unit patients, both with a high risk of falling.

Since the results for NPVs (98.9%±0.7%) indicate that with these algorithms there is a high probability that when an event is not detected as a fall it is not really a fall, the PPVs (19.3%±9.7%) are low, i.e., there is a low probability that when a fall is detected it is really a fall. This means that some events incorrectly detected as falls are activities of daily living.

Furthermore, since the number of real-world falls (29) is small compared with the total number of time-windows tested (1170), the SP is affected by these differences and does not provide useful information for the evaluation of the algorithms (e.g., Bourke3 has 97% specificity i.e. 39 false positives). From a more practical point of view, if the fall detector is connected to a tele-alarm system, the robustness of the fall detection algorithm should be evaluated in terms of high sensitivity and small number of false alarms generated. For example, consider a recording of 48 *h*, i.e. 2880 time windows of 60 *s*. If the algorithm incorrectly detects 100 ADL as falls, the SP will be 96%. This is an acceptable value for a test but if we imagine that the fall detector could trigger an alarm when a fall is detected, 100 false positive results within 48 *h* means that about 2 false alarms per hour are generated.

The weaknesses of the tested algorithms enable us to understand certain complex aspects of a fall but could also be a starting point for future development of an accurate fall detector. The tested algorithms set a fixed threshold for features extracted by accelerometer signals but are tested on individuals with different mass, age, clinical history and diseases. These factors could affect the accelerometer data and the algorithms could fail; a fixed threshold may not be the optimal strategy compared to a subject-specific threshold. Inertial sensors-based fall detection algorithms could be designed not only to automatically detect a fall but also to provide additional information regarding direction of falls in order to better understand injuries and to offer a prevention intervention. Since results are related to the thresholds provided by the authors, the performance of the tested algorithms could be optimized by using the Receiving Operating Characteristic (ROC) in order to identify the threshold with the best trade-off between sensitivity and specificity. Despite this, the results presented in this paper showed the importance of testing algorithms in real-world situations. The main limitation of the study is that the recorded real-falls were from a rare disease population, but conclusions may be generalized to the older population at large. Moreover, the tested algorithms are based only on waist or trunk accelerometer measurements and therefore did not represent an exhaustive set of all published fall detection algorithms.

The development of a larger shared real-world fall database should provide additional data and deepen our knowledge of the fall process in general. The FARSEEING European project, which started in January 2012, aims to build the world's largest fall repository of long-term analysis of the behavioral and physiological data collected using smartphones, wearable and environmental sensors. This project could provide the necessary data to design an accurate, portable and high-performance fall detector and a more valid model of falling. The present paper represents a preliminary study in this direction.

## Supporting Information

Table S1 Brief review of the main fall detection algorithms.
(DOCX)
