# Supplementary material for: Evaluation of Accelerometer-Based Fall Detection Algorithms on Real-World Falls
Source: PLoS One. 2012 May 16;7(5):e37062. doi: 10.1371/journal.pone.0037062 (PMC3353905; doi:10.1371/journal.pone.0037062)
Supplement: Table S1 — Brief review of the main fall detection algorithms. (DOCX) [file pone.0037062.s001.docx]

| **Authors** | **Parameters** | **Thresholds** | **Algorithm’s rationale** |
| --- | --- | --- | --- |
| ***Chen et al.*** | $\mathbf{SV} =\sqrt{\mathbf{A}_{x}^{2}+\mathbf{A}_{y}^{2}+\mathbf{A}_{z}^{2}}$  ^(1)^ | >3 g  Set arbitrarily based on empirical data (>20°) | IMPACT DETECTION  +  CHANGE IN ORIENTATION |
| ***Kangas et al. (1a)*** |   $\mathbf{SV} =\sqrt{\mathbf{A}_{x}^{2}+\mathbf{A}_{y}^{2}+\mathbf{A}_{z}^{2}}$ $Posture=\bar{\mathbf{A}}_{x,LPF}$^(2)^ | >2 g  <0.5 g | IMPACT DETECTION  +  POSTURE MONITORING |
| ***Kangas et al. (1b)*** | $\mathbf{SV}_{D} =\sqrt{\mathbf{A}_{x,HPF}^{2}+\mathbf{A}_{y,HPF}^{2}+\mathbf{A}_{z,HPF}^{2}}$^(3)^ $Posture=\bar{\mathbf{A}}_{x,LPF}$  | >1.7 g  <0.5 g | IMPACT DETECTION  +  POSTURE MONITORING |
| ***Kangas et al. (1c)*** | $\begin{matrix} SV_{MaxMin}(k)=\max(SV(k:k+0.1*fs))-\min(SV(k:k+0.1*fs))\text{ } \\ \text{ }k=1,..n-0.1*fs \end{matrix}$ $Posture=\bar{\mathbf{A}}_{x,LPF}$   | >2 g  <0.5 g | IMPACT DETECTION  +  POSTURE MONITORING |
| ***Kangas et al. (1d)*** | $\mathbf{X}_{2}=\frac{\mathbf{SV}^{2}-\mathbf{SV}_{D}^{2}-G}{2G}\text{ }\left(G =1g\right)$ $Posture=\bar{\mathbf{A}}_{x,LPF}$   | >1.5 g  <0.5g | IMPACT DETECTION  +  POSTURE MONITORING |
| ***Kangas et al. (2a)*** | $\mathbf{SV} =\sqrt{\mathbf{A}_{x}^{2}+\mathbf{A}_{y}^{2}+\mathbf{A}_{z}^{2}}$ (start of fall) $\mathbf{SV}$ $Posture=\bar{\mathbf{A}}_{x,LPF}$    | <0.6 g  >2 g  <0.5g | START OF FALL  +  IMPACT DETECTION  +  POSTURE MONITORING |
| ***Kangas et al. (2b)*** | $\mathbf{SV}$ (start of fall) $\mathbf{X}_{2}$ $Posture=\bar{\mathbf{A}}_{x,LPF}$    | <0.6 g  >1.5 g  <0.5g | START OF FALL  +  IMPACT DETECTION  +  POSTURE MONITORING |
| ***Kangas et al. (3a)*** | $\mathbf{SV}$ (start of fall) $v_{0}=\int_{t_{start\text{ }fall}}^{t_{impact}} SV(\tau)d\tau$ $\mathbf{SV}$ $Posture=\bar{\mathbf{A}}_{x,LPF}$      | <0.6 g  >0.7 m/s  >2 g  <0.5g | START OF FALL  +  VELOCITY  +  IMPACT DETECTION  +  POSTURE MONITORING |
| ***Kangas et al. (3b)*** | $\mathbf{SV}$ (start of fall) $v_{0}=\int_{t_{start\text{ }fall}}^{t_{impact}} SV(\tau)d\tau$ $\mathbf{X} _{2}$ $Posture=\bar{\mathbf{A}}_{x,LPF}$      | <0.6 g  >0.7 m/s  >1.5 g  <0.5g | START OF FALL  +  VELOCITY  +  IMPACT DETECTION  +  POSTURE MONITORING |
| ***Bourke et al. (1(a))*** | $\mathbf{SV} =\sqrt{\mathbf{A}_{x}^{2}+\mathbf{A}_{y}^{2}+\mathbf{A}_{z}^{2}}$ | >UFT 1.79 g | IMPACT DETECTION |
| ***Bourke et al. (1(b))*** | $\mathbf{SV} =\sqrt{\mathbf{A}_{x}^{2}+\mathbf{A}_{y}^{2}+\mathbf{A}_{z}^{2}}$ | <LFT 0.73 g | IMPACT DETECTION |
| ***Bourke et al. (2)*** | $\mathbf{SV} =\sqrt{\mathbf{A}_{x}^{2}+\mathbf{A}_{y}^{2}+\mathbf{A}_{z}^{2}}$ $orientation=\mathbf{A}_{x}$   | >1.79 g $-0.5g<A_{x}<0.5g$  | IMPACT DETECTION  +  POSTURE DETECTION |
| ***Bourke et al. (3)*** | $v_{0}=\int_{t_{start\text{ }fall}}^{t_{impact}} SV(\tau)d\tau$ $\mathbf{SV} =\sqrt{\mathbf{A}_{x}^{2}+\mathbf{A}_{y}^{2}+\mathbf{A}_{z}^{2}}$ $\vartheta(t)=\cos^{-1}\left(\frac{\mathbf{g}_{SEG}(t)\bullet\mathbf{g}_{REF}}{\left\vert\mathbf{g}_{SEG}(t)\right\vert\bullet\left\vert\mathbf{g}_{REF}\right\vert}\right)\frac{180}{\pi}$     | <-0.7 m/s  >UFT 2.8 g  <LFT 0.65 g  60° | VELOCITY  +  IMPACT DETECTION  +  POSTURE |

^(1)^An angle of change can be estimated using the dot product of the acceleration vectors before a fall and after, where the vectors are from averaging over 1-second windows. ^(2-3)^Accelerometric data were low-pass (LPF) or high-pass (HPF) filtered ($f_{c}=0.25Hz$) with a digital second order Butterworth filter.
